# Complex variation in habitat selection strategies among individuals driven by extrinsic factors

**DOI:** 10.1002/ece3.2764

**Published:** 2017-02-15

**Authors:** Edward J. Raynor, Hawthorne L. Beyer, John M. Briggs, Anthony Joern

**Affiliations:** ^1^ Division of BiologyKansas State UniversityManhattanKSUSA; ^2^ ARC Centre of Excellence for Environmental DecisionsThe University of QueenslandBrisbaneQldAustralia; ^3^ Present address: School of Natural ResourcesUniversity of NebraskaLincolnNEUSA

**Keywords:** *Bison bison*, climatic variability, forage maturation hypothesis, forage quality–quantity tradeoffs, Konza Prairie, resource selection plasticity, step selection, weather‐induced resource variation

## Abstract

Understanding behavioral strategies employed by animals to maximize fitness in the face of environmental heterogeneity, variability, and uncertainty is a central aim of animal ecology. Flexibility in behavior may be key to how animals respond to climate and environmental change. Using a mechanistic modeling framework for simultaneously quantifying the effects of habitat preference and intrinsic movement on space use at the landscape scale, we investigate how movement and habitat selection vary among individuals and years in response to forage quality–quantity tradeoffs, environmental conditions, and variable annual climate. We evaluated the association of dynamic, biotic forage resources and static, abiotic landscape features with large grazer movement decisions in an experimental landscape, where forage resources vary in response to prescribed burning, grazing by a native herbivore, the plains bison (*Bison bison bison*), and a continental climate. Our goal was to determine how biotic and abiotic factors mediate bison movement decisions in a nutritionally heterogeneous grassland. We integrated spatially explicit relocations of GPS‐collared bison and extensive vegetation surveys to relate movement paths to grassland attributes over a time period spanning a regionwide drought and average weather conditions. Movement decisions were affected by foliar crude content and low stature forage biomass across years with substantial interannual variation in the magnitude of selection for forage quality and quantity. These differences were associated with interannual differences in climate and growing conditions from the previous year. Our results provide experimental evidence for understanding how the forage quality–quantity tradeoff and fine‐scale topography drives fine‐scale movement decisions under varying environmental conditions.

## 1 Introduction

Many animals respond to environmental heterogeneity through selectivity in their choice of habitats to best fulfill basic requirements such as the need to feed, reproduce, and rear offspring (Brown et al. 1999; Morris, 2003; Mueller & Fagan, 2008). Movement enables animals to mediate tradeoffs in life‐history requirements arising from the heterogeneous distribution of resources (Nathan, 2008). Integrating complex and dynamic interactions between intrinsic and extrinsic processes and their interactions that drive movement and distribution of individuals in a population remains an important challenge. For large mammalian grazers, the spatial distribution of forage and its associated nutritive value are fundamental components underlying foraging behavior, resource selection, and landscape‐level distribution (Bailey et al., 1996; Fynn, 2012; Prins & van Langevelde, 2008; Senft et al., 1987; Spalinger & Hobbs, 1992). In addition, identifying the determinants of large grazer distribution is important for the effective management of both rangelands and the populations of grazers inhabiting them (Archer & Smeins, 1991; Dale et al., 2000; Fynn, 2012). Understanding how ecologically significant resources such as forage biomass and forage nutrient content affect grazer resource selection is necessary for informing management strategies (Senft, Rittenhouse, & Woodmansee, 1985), particularly in areas experiencing reduced growing season precipitation and increasing ecosystem sensitivity due to climate change (Briske et al., 2015; Knapp et al., 2015).

Animal movement is influenced by a wide variety of intrinsic and extrinsic factors relating to static and dynamic environmental conditions and the state of the animal (Bailey et al., 1996; Beyer et al., 2010; Owen‐Smith, 2002). Although considerable work has been carried out in understanding the role of static conditions on movement, less is understood about how animal movement strategies vary among individuals and years in response to both within‐ and between‐season changes in environmental conditions. Behavioral flexibility could play an essential role in determining to what extent fitness of individual grazers and population dynamics is affected by climate change impacts on rangelands over the coming decades.

Optimal foraging theory predicts that animal distribution should reflect the distribution of energy/nutrient‐rich resources on a landscape (MacArthur & Pianka, 1966), where ungulates exploit forage quality in efforts to maximize intake rate (Albon & Langvatn, 1992; Fryxell, Greever, & Sinclair, 1988; McNaughton, 1985). However, energy and nutrient intake is not simply a function of forage quality, but of tradeoffs between forage quality and quantity (Fryxell, 1991; Hebblewhite, Merrill, & McDermid, 2008). An inverse correlation between forage quantity and forage processing constraints (i.e., digestibility and gut passage rates; Gross, Shipley, Hobbs, Spalinger, & Wunder, 1993; Spalinger & Hobbs, 1992) creates a tradeoff for grazing herbivores (Fryxell, 1991; McNaughton, 1979; Van der Wal et al., 2000). Foraging ruminants can maximize their short‐term instantaneous intake rate of digestible energy by consuming large plants that result in rapid satiation (Gross et al., 1993; Spalinger & Hobbs, 1992). Alternately, foragers can maximize their daily intake of digestible energy/protein by foraging on small and/or immature plants (Wilmshurst & Fryxell, 1995), which demand more time (cropping) to reach satiation, but ultimately provide more digestible energy/protein due to their higher digestibility than large plants (Bergman, Fryxell, Gates, & Fortin, 2001; Wilmshurst, Fryxell, & Hudson, 1995). Because forage quality and digestibility decline with plant maturation, grazers are predicted to select for low‐to‐intermediate biomass to maximize energy/protein intake by tracking high‐quality forage (Bischof et al., 2012; Fryxell, 1991; Hebblewhite et al., 2008; McNaughton, 1979; Merkle et al., 2016; Wilmshurst & Fryxell, 1995). This is the basis of the forage maturation hypothesis (Fryxell, 1991), which posits that foragers achieve the most energetic/nutritional gain by feeding at sites where biomass is at low to moderate levels. Recursive grazing can facilitate enhancement of forage quality that can guide restricted space use as long as regrowth is possible (Arsenault & Owen‐Smith, 2002; Augustine & Springer, 2013; McNaughton, 1976, 1986; Raynor et al. 2016). To date, few studies have assessed the role of this dynamic forage quality–quantity tradeoff in guiding broad‐scale grazer movement (but see Hebblewhite et al., 2008), and, to our knowledge, even fewer have evaluated how extrinsic environmental factors mediate these decisions.

Because grazing systems are exceedingly common in both the United States (61% of all land surface) and the world (70%; Fuhlendorf and Engle 2001), understanding how extrinsic factors such as local climate dictate grazer land use is important for predicting the effects of climate change at global scales. Efforts to restore large grazing herbivores to their historic range would benefit from evaluations of the effects of interannual variability of resources on animal movement (Kuemmerle et al., 2011; Steenweg, Hebblewhite, Gummer, Low, & Hunt, 2016). Moreover, changes in movement patterns can be used as behavioral indicators of stressful conditions before the consequences for survival and reproduction are manifested (Owen‐Smith & Cain, 2007). A broader understanding of the relationships between local climate conditions and habitat selection is important because successful conservation and management must be based on rigorous understanding of the impact of environmental factors on the ability of animals to adapt behaviorally to changing environmental conditions (Matthiopoulos, Hebblewhite, Aarts, & Fieberg, 2011; Matthiopoulos et al., 2015).

In this study, we relate detailed movement trajectories of large grazing herbivores, matriarchal female bison (*Bison bison bison*), to fine‐scale grassland attributes over seven growing seasons characterized by average to below‐average forage production in a tallgrass prairie (Konza Prairie Biological Station [KPBS]). We use fine‐scale, mechanistic movement models to quantify interannual variation in both movement and habitat selection, and use these models to evaluate how bison respond to the forage quantity–quality tradeoff and how these strategies change among years with distinctly different climate conditions. We incorporate two ecologically significant resources, forage biomass and forage nitrogen content, projected across the landscape at high temporal (biweekly) and spatial (10 m^2^) resolutions based on empirically parametrized models. Our dynamic vegetation modeling incorporated vegetation responses to prescribed burning and local weather conditions. The movement modeling identifies large grazer interactions with prescribed burning‐ and local weather‐induced variation in forage quality and quantity, both of which are integral underlying ecological process for maintenance of grassland heterogeneity (Fuhlendorf and Engle 2001; Fynn, 2012). Because our study spanned growing seasons of varying forage availability, we were able to evaluate variation in large grazer resource selection under varying environmental conditions and provide insight into how individuals respond to environmental change. Quantifying the mechanisms underlying animal movements and distribution in the context of environmental and climate change is integral to understanding ecosystem function and restoring natural processes (Archer & Smeins, 1991; Wiens, Stralberg, Jongsomjit, Howell, & Snyder, 2009) and could provide the quantitative basis for projecting future ecological scenarios (Coreau, Pinay, Thompson, Cheptou, & Mermet, 2009) and reducing human–wildlife conflicts (Naughton‐Treves, 1998).

Using a mechanistic framework that includes (1) empirically based estimates of forage quality and quantity and (2) a conditional resource selection analysis that allows simultaneous estimation of resource selection and movement, we were able to predict how large grazer movement decisions relate to grassland attributes in a nutritionally heterogeneous landscape. Because the net energy deficit for animals departing winter conditions (Parker et al. 2009) is likely to be greatest following years of low forage production, we predict (*a*) selection for forage quantity will be highest in growing seasons following seasons with poor forage production conditions. Rather than mobilizing reserves to meet shortfalls in nutritional and caloric maintenance (Owen‐Smith, 2002; Shrader, Owen‐Smith, & Ogutu, 2006), large grazers can compensate for low nutrient availability by consuming a greater quantity of forage irrespective of nutritive value (Illius, Duncan, Richard, & Mesochina, 2002; Laca, Ungar, & Demment, 1994). We expect large grazers to select foraging habitats with higher forage biomass than other habitats along their movement path when past growing season conditions were poor. In contrast, during periods of high forage production when nutrients are less concentrated in leaf tissue than low forage production years (Jones & Coleman, 1991) we predict (*b*) selection for forage with high nutritional value will be consistently high. In the tallgrass prairie landscape, habitat containing highly accessible foliar protein is associated with low vegetation stature (Schimel et al., 1991), resulting from recursive grazing of grass regrowth (Raynor et al. 2016). Adequate forage protein content is required to keep the rumen microbial system functional during critical times of the year (Faverdin, 1999; Van Soest, 1994); therefore, in efforts to meet the demands of food processing and digestion we predict (*c*) bison will generally select areas containing high foliar protein content and low forage biomass.

It is well known that large grazers in temperate systems use topographic characteristics of the landscape to meet basic maintenance requirements, such as regulating thermal balance (Mysterud, Langvatn, Yoccoz, & Stenseth, 2001; Street et al., 2016), yet most studies do not identify the topographic resources driving interannual variability in movement patterns as such studies are usually short term (e.g., Senft et al., 1985). However, how large grazer selection for these landscape features may vary from year to year in response to environmental change is in need of study. We test the (*d*) prediction that in years with high growing season temperatures, selection for topographic attributes will not be strong drivers of habitat selection. During periods of very high air temperature, grazers seek out thermal refugia and water resources in low‐lying riparian areas (Allred et al., 2013). During years of high growing season temperatures, we expect bison to use lower elevations compared to all available locations and areas of nonsoutherly aspect as these locations contain lowland habitat in this study area with higher soil moisture availability for promoting postfire regrowth compared to less‐productive uplands (Hopcraft, Olff, & Sinclair, 2010; Knapp et al., 1993; Nippert et al., 2011).

## 2 Methods

### 2.1 Study area and bison population

**Figure 1:**
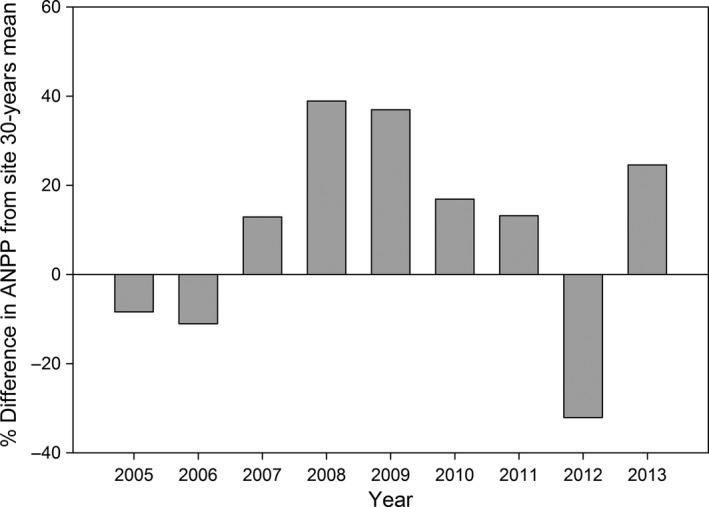
Difference of annual net primary productivity (ANPP) from 30‐year mean during 2005–2013 at Konza Prairie Biological Station, Manhattan, Kansas, USA

Our study took place from 2007 to 2013 at the Konza Prairie Biological Station (KPBS), a 3,487‐ha native tallgrass prairie preserve located in the Flint Hills grassland near Manhattan, Kansas (USA) (39°05′N, 96°35′W) (Knapp, Briggs, Blair, & Turner, 1998). Vegetation is mostly tallgrass prairie dominated by C_4_ grasses (*Andropogon gerardii*,* Schizachyrium scoparium*,* Sorghastrum nutans*, and *Panicum virgatum*) along with a diverse mixture of warm‐ and cool‐season graminoids. Average monthly temperatures range from −2.7°C (January) to 26.6°C (July). Average annual precipitation is ~835 mm, with 75% falling during the growing season. During winter, snow does not accumulate and grazers are able to consume forage unhindered by snow cover. Mean growing season temperature was above the 30‐year study area mean during 2010–2012 and a drought occurred from mid‐summer of 2011 through the entire growing season in 2012 which caused the annual net primary productivity (ANPP) to be well below the 30‐year study area mean (Figure 1, Knapp et al., 2015). In 2007–2009 and 2013, total growing season precipitation and ANPP were near or above the recorded mean for the study area.

**Figure 2:**
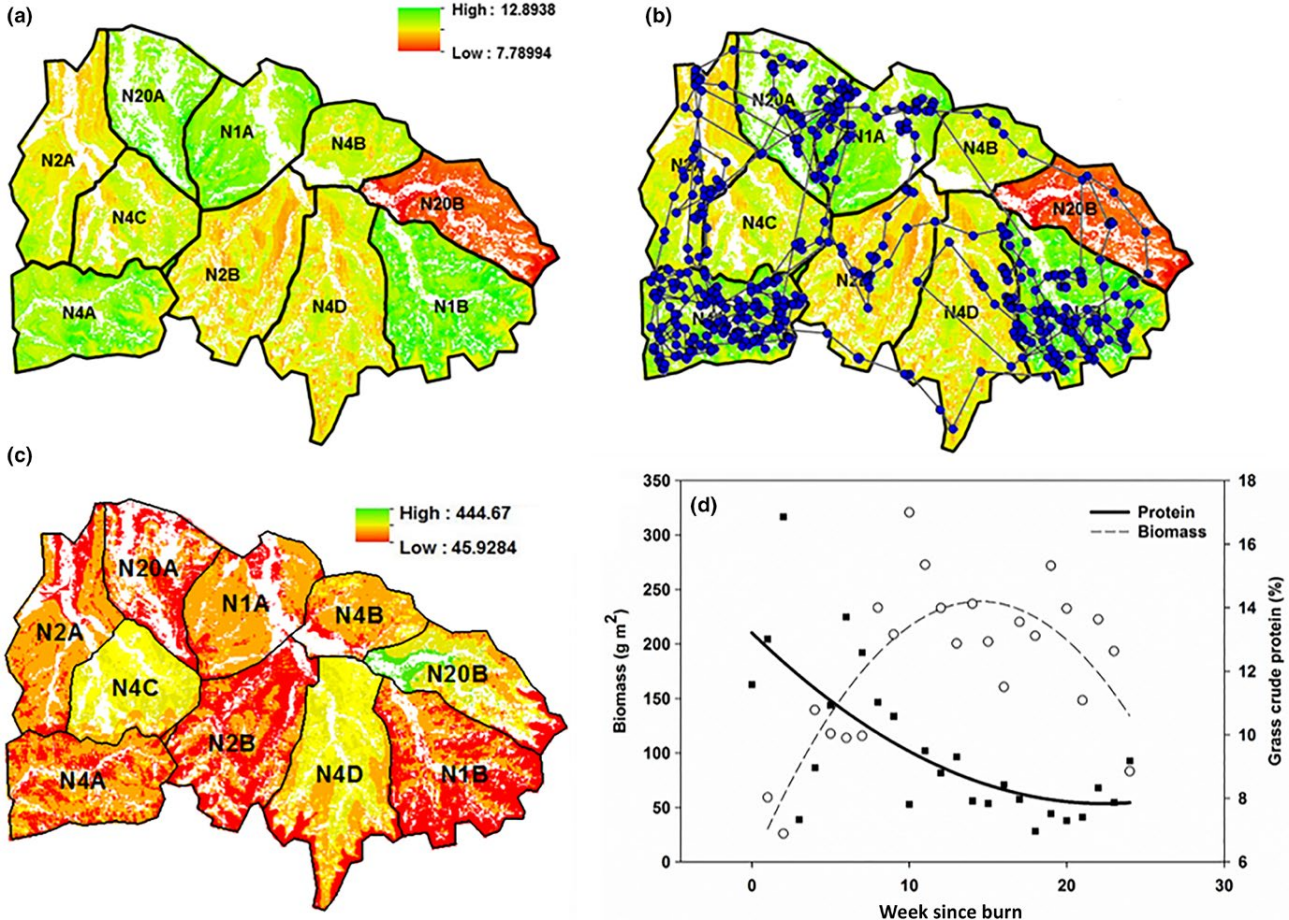
Map showing the prediction of (a) grass crude protein content (10 m resolution) in the Konza Prairie Biological Station bison enclosure obtained by the application of the Random Forest model for May 2012, (b) with movement path of bison #W674 over grass protein availability for May 2012 as an example of bison responses, (c) map showing the prediction of forage biomass (10 m resolution), and (d) relationship between time since fire and forage resources for watersheds that burned in spring

**Figure A1:**
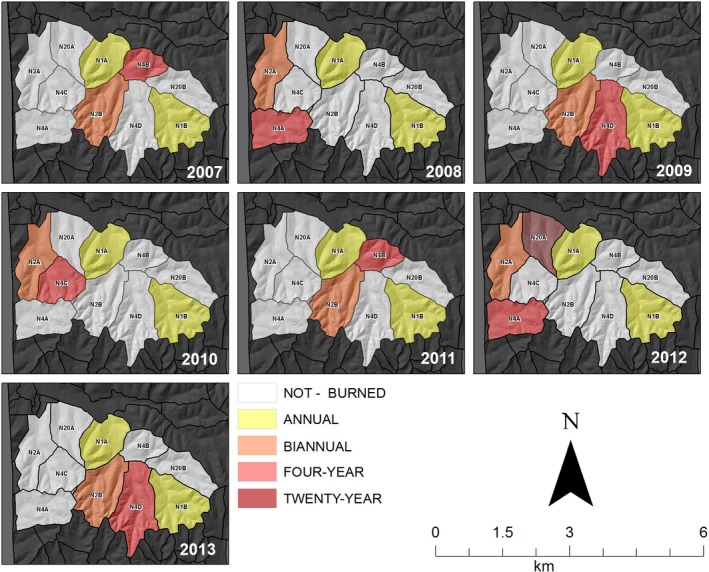
Map of bison enclosure at Konza Prairie Biological Station, Manhattan, KS, USA showing topographic relief and watersheds burned each year. Watersheds are labeled according to fire frequency (1, 2, 4, 20 years between burns); all watersheds (*x*′ = ~100 ha, range = 80–200) included here are part of the bison unit labeled as N, native grazer. A watershed label indicates replicate number (A–D). For example, N04D is replicate D of a bison‐grazed watershed (N) burned every 4 years

Bison at KPBS have free access to 10 experimental watersheds over approximately ~970 ha subjected to 1‐, 2‐, 4‐, and 20‐year burn‐interval treatments within a fenced enclosure (Figure A1; herd history and management is described in supplemental material). All prescribed management burns are conducted in the spring (mid‐March to early May). Foliar protein content of graminoids is slightly higher in burned watersheds (Raynor, Joern, & Briggs, 2015) with peak protein availability occurring soon after prescribed burns (~early May; curvilinear regression; *F*_2,24_ = 10.52, *R* ^2^=.44, *p *=.001; Figure 2d). Forage biomass of burned watersheds is lower than unburned watersheds in spring due to recurrent grazing (Raynor et al., 2015) with peak biomass availability generally occurring mid‐summer in burned watersheds (curvilinear regression; *F*_2,22_ = 15.90, *R* ^2^ = .58, *p* < .0001, Figure 2f) and unburned watersheds in the spring (*F*_2,22_ = 0.54, *R* ^2^ = .05, *p *= .58; Figure 2f). During the growing season, bison preferentially use recently burned sites (40% of available area) over those not burned during the spring burning period (60% of available area; Vinton, Hartnett, Finck, & Briggs, 1993), then move to unburned sites in the dormant season (Raynor, 2015; Raynor et al., 2015).

Adult female bison were tracked using Telonics TGW‐3700 GPS collars during 2007–2013. Four individuals were tracked in 2007, seven in 2008–2009, 11 in 2010, 14 in 2011, 13 in 2012, and 11 in 2013; totaling 67 individual‐years among 20 individuals. We used four‐hour collar fixes collected from 1 April to 30 September in our analyses. Estimates indicate that collared animals are often accompanied by roughly 30–40 individuals (E. J. Raynor, unpublished data), a value that fluctuates somewhat depending on whether the herd is coalesced or fragmented at the time. Collars were fitted or replaced annually at the end of the growing season, using the same individuals in consecutive years when possible.

### 2.2 Forage quality–quantity dynamics

The quality of the forage was estimated from foliar nitrogen concentrations of grasses measured at 1,039 locations between the 2011 and 2013 growing seasons and opportunistically distributed throughout the different watershed burn types at KPBS. The aboveground grass biomass was clipped in 25 × 25 cm plots at each of the 1,039 locations (pooling all graminoid species) and air‐dried, ground to a 1‐mm particle size, and analyzed by Dairyland Laboratories (Arcadia, Wisconsin, USA) on a Foss model 5000 Near Infra‐Red (NIR) spectrophotometer (Foss, Hillerød, Denmark). Crude protein (%) was estimated as % N in plant tissue * 6.25 (Jones, 1941).

The quantity of forage was estimated at 16,792 locations that were opportunistically located between the 2011 and 2013 growing seasons and distributed throughout different watershed burn types at KPBS. Total dry plant biomass (*B*; g/m^2^) was estimated using a calibrated pasture disk meter that measured the height (cm) to which a plastic disk of constant weight could be supported as it settled on top of the canopy (Vartha & Matches, 1977). Height was related to total plant biomass by regressing pasture meter readings on plots that were subsequently harvested to determine dry biomass, leading to the following regression models: BIOMASS = 2.40HEIGHT + 3.70, *R* ^2^ = .85, *p* < .0001, *n* = 35 for 2012 [a drought year] and BIOMASS = 3.78HEIGHT + 6.18, *R* ^2^ = .63, *p* < .0001, *n* = 55 for 2013 [a normal year for precipitation]. The calibration for 2013 was used to estimate herbaceous biomass at sites from 2011 when ANPP was similar to 2013.

We used random forest (RF) regression models (Liaw & Wiener, 2002) to estimate grass nitrogen content and herbaceous biomass in watersheds as a function of cumulative precipitation in that year, time since burn, and site topography. The response variable was predicted from the combination of all regression trees (trees = 1,000, terminal node size = 5). This approach performs well when modeling nonlinear relationships between predictors and the response and accommodates complex interactions among predictors (Bohrer, Beck, Ngene, Skidmore, & Douglas‐Hamilton, 2014). These model properties are important for modeling forage quality and quantity relationships across space because nutritive and structural values of plants are spatially heterogeneous (e.g., along environmental gradients). Interactions between spatial (e.g., topography) and temporal (e.g., cumulative precipitation and time since burn) predictors can be effectively incorporated into the model (Prasad, Iverson, & Liaw, 2006). The topographic characteristics assigned to each site sampled during the growing seasons of 2011–2013 included the following: the sine and cosine of aspect (radians), slope (degrees), and scaled elevation (m) extracted from an existing digital elevation model (DEM, with spatial resolution of 2 × 2 m; ~333–443 m a.b.s.l). Cumulative daily precipitation (mm) collected on site and the number of days since the sampling area burned was assigned to each sampling event. Accounting for topographic variation and meteorological events are important parameters for determining aboveground herbaceous biomass at KPBS (Briggs & Knapp, 1995). The number of times the watershed burned since 1980 and type of burn schedule assigned to the watershed, and if the watershed burned in a particular year were additional predictors incorporated into the RF models.

We trained the model on a randomly selected set of data comprising 33% of the sites and withheld the remaining 67% to test model performance. Performance was assessed using the root mean squared error of log‐transformed response variable. This validation procedure was repeated 10 times, and model performance was characterized using the average root mean squared error from the 10 random validation datasets. RF models were fit using the library *randomForest* (Liaw & Wiener, 2014) in R (R Development Core Team 2014).

The grass nitrogen and herbaceous biomass models described above were used to project grass nitrogen and herbaceous biomass across a 10‐m grid of points throughout the bison enclosure, excluding points within a 1 m radius of known shrub cover identified from a 1 × 1 m resolution raster map from the 2011 growing season (Ling, Goodin, Mohler, Laws, & Joern, 2014). For this extrapolation, the model was trained on the entire 2011–2013 dataset (as opposed to the 33% used for model validation described in the previous section). Year was not used as a predictive variable in the RF model, instead, cumulative precipitation since 1 March and time since burn were substituted for the temporal aspect of the projection model. This allowed us to predict spatial and temporal coverage of forage quality and quantity across the entire bison enclosure at biweekly intervals from 1 April to 1 October in the 2007–2013 growing seasons. Biweekly raster projections of grass crude protein content and herbaceous biomass were generated across the entire enclosure for use in bison movement modeling (Figure 2a,c).

### 2.3 Modeling effects of environmental variables on movement

We modeled movement patterns in relation to forage resource variability driven by landscape‐level disturbance arising from fire frequency, local weather, and topographic variables. Extrinsic biases to bison movement were evaluated by comparing observed and random steps through the heterogeneous landscape based on a case–control design (Boyce et al., 2003). We explicitly considered landscape characteristics that animals would have been likely to encounter along their path (a step selection function; Fortin et al., 2005). We assessed collinearity among variables using Pearson's correlation coefficients.

We model animal movement and habitat selection using the framework of Beyer et al. (2016), which defines the probability that an animal moves from location *a* to location *b* (a “step”) in a given time interval and conditional on habitat covariates, *X*, at location *b* to be:

(1) $$f\left(b|a,X\right)=\frac{\phi \left(a,\;b,\;\Delta t;\;\theta \right)\;\omega \left(X_{b};\;\beta \right)}{\int_{c\in D}\phi \left(a,\;c,\;\Delta t;\;\theta \right)\;\omega \left(X_{c};\;\beta \right)dc},$$

where ϕ(*a*,* b*, Δ*t*; θ) is a two dimensional probability density function describing the probability of the location of the next location after Δ*t* as a function of the current location at the center of that distribution (this is also sometimes referred to as a redistribution kernel or habitat‐independent movement kernel), and ω(*X*) is the resource selection probability function and *X* is a matrix of habitat covariates (including a column of 1's representing the intercept term; Lele & Keim, 2006). Here, ϕ(*a*,* b*, Δ*t*; θ) is a bivariate normal distribution with equal variance in the *x* and *y* dimensions determined by the parameter θ, and ω is a logistic model with coefficients β representing the habitat preferences. It is also possible to use alternative distributions for ϕ (*a*,* b*, Δ*t*; θ) s that incorporate directional persistence (e.g., Avgar, Potts, Lewis, & Boyce, 2016; Forester, Im, & Rathouz, 2009). Habitat covariates included elevation (m), slope (degrees), cosine of aspect (radians), grass crude protein content (% CP), herbaceous biomass content (g/m^2^), and the interaction of foliar protein content and biomass, all of which were raster format data sets with a spatial resolution of 10 × 10 m. Specifically, the habitat selection model was as follows:

$$\begin{array}{cc} \text{logit}\left(\omega (Xb;\beta )\right)= & \text{exp}(\beta_{1}\text{ELEV}+\beta_{2}\text{SLOPE}+\beta_{3}\text{Cos(ASPECT)}\\ & +\beta_{4}\text{PROTEIN}+\beta_{5}\text{BIOMASS}\\ & +\beta_{6}\text{PROTEIN}*\text{BIOMASS}). \end{array}$$

The numerator of Equation (1) is normalized by the denominator, integrated over all locations, *c*, with the spatial domain, *D*. The denominator can be approximated by sampling the domain, hence each observed step was paired with 100 random steps in a case‐controlled “step selection function” design (Fortin et al., 2005). We simultaneously estimated the habitat‐independent movement kernel and habitat preference by fitting *f*(*b*|*a*,* X*) (eqn (1)) to the location data (see Beyer et al., 2016 for details) for each individual in each year using the “*optim*” function in R (version 3.0.2, R Development Core Team 2014). Confidence intervals for the parameter estimates were calculated from the Hessian matrix (±1.96 times the square roots of the diagonal elements of the covariance matrix).

### 2.4 Data analyses

The maximum‐likelihood estimates for each of the habitat selection coefficients for each individual in each year were used as the dependent variables in subsequent analyses to evaluate how selection varied among years and in relation to individual reproductive status and local weather conditions (i.e., previous‐year forage production and current‐year growing season temperature). We adopted a linear mixed‐effects (LME) model framework using the R library *lme4* (Bates, Mächler, Bolker, & Walker, 2015) with individual identifier (eartag) as the random effect to account for the fact that multiple observations from a single animal among years are not independent (range: 2–7 years, median: 3 years). For the year term included in the LME model, we used the glht function in the R library *multcomp* (Hothorn, Bretz, & Westfall, 2013) to calculate Tukey's honest significant differences (HSD) among years in habitat selection coefficients. All comparisons were considered statistically significantly different when *p* < .05. Kenward–Roger's approximation was used to calculate effective degrees of freedom of a linear combination of independent sample variances (Kenward & Roger, 1997). Next, we evaluated whether selection for grassland attributes was related to previous growing season forage production and growing season temperature and whether selection differed by individual reproductive status. Because the animals studied here were sexually mature adult females (*x̄* ± SD: 10 ± 2.97 years old) with known reproductive status, we tested whether selection or foliar protein, forage biomass, elevation, slope, and cosine of aspect differed between females with or without calves. Calf–mother pairs were identified by behavioral observations such as suckling and proximity in spring and soon after the annual roundup, ensuring that female bison GPS locations prior to autumn roundup of mothers with spring‐born calves represent valid calf–mother pairs, thereby reliably determining the reproductive status of the female. Previous‐year annual net primary productivity (ANPP) levels are derived from mean values of live tissue clipped at nongrazed, study plots in nongrazed watersheds, 1D, 04B, 20B (LTER dataset: PAB011, https://lternet.edu/sites/knz) during the end of the previous growing season (~15 September, one measure per year). Using LME with individual identifier as a random effect, mean previous‐year ANPP (0.1 g m^2^) from these sampling plots were regressed against habitat selection coefficients to assess the effect of past growing season forage production on current‐year habitat selection. In addition, we assessed how growing season temperature, an average of temperature (°C) at KPBS headquarters from April to October, related to current‐year habitat selection.

## 3 Results

### 3.1 Forage quality and quantity

**Figure A2:**
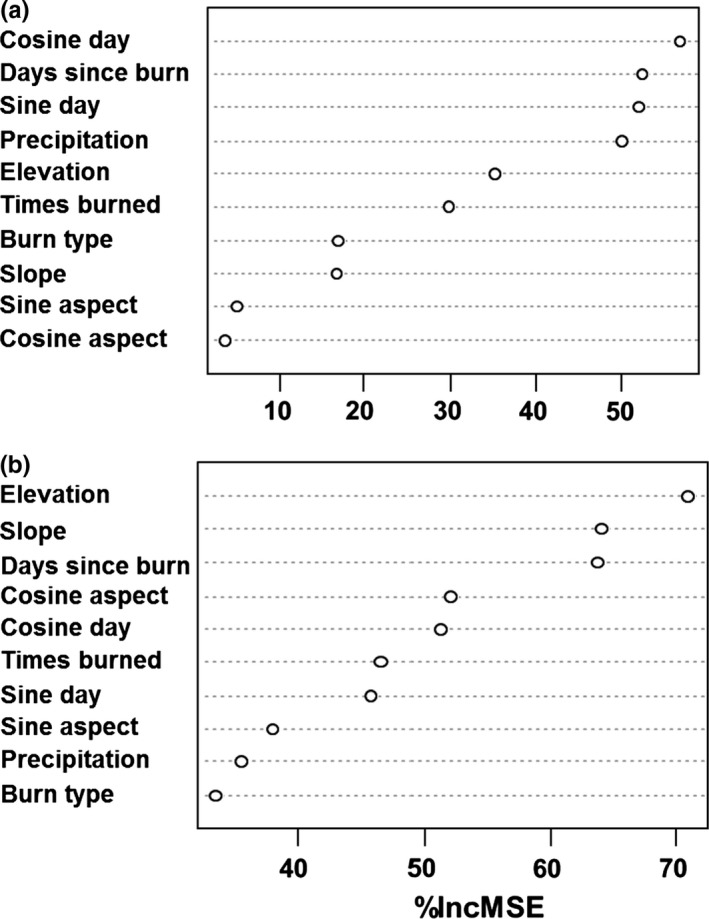
Variable importance plots for (a) grass nitrogen content and (b) herbaceous biomass content generated by the random forest algorithm included in the *randomForest* package for R software. The plot shows the variable importance measured as the increased mean square error (%IncMSE), which represents the deterioration of the predictive ability of the model when each predictor is replaced in turn by random noise. Higher %IncMSE indicates greater variable importance. Variables include cosine of day, days since last burn of watershed, sine of day, cumulative precipitation since March 1, elevation (m; scaled), number of times burned since 1980, burn type [frequent, infrequent, not burned that year], slope (degrees), sine of aspect (radians), and cosine of aspect (radians)

For the training dataset, the RF model explained a large proportion of the variance of the foliar protein content (pseudo *R* ^2^ = .72) and forage biomass (pseudo *R* ^2^ = .49). The root mean square error averaged across the 10 random validation datasets was 1.47 for forage biomass (*n* = 5541 samples) and 1.03 for foliar protein (*n* = 343 samples). Only a few of the descriptors contributed substantially to the estimation of crude protein content, namely elevation, slope, and days since burn (Figure A2a). For forage biomass, descriptors that contributed substantially to its estimation included: cosine of Julian day (rescaled to 0‐2π radians), day since burn, sine of day, and cumulative precipitation (mm) (Figure A2b).

### 3.2 Bison habitat preference

**Figure 3:**
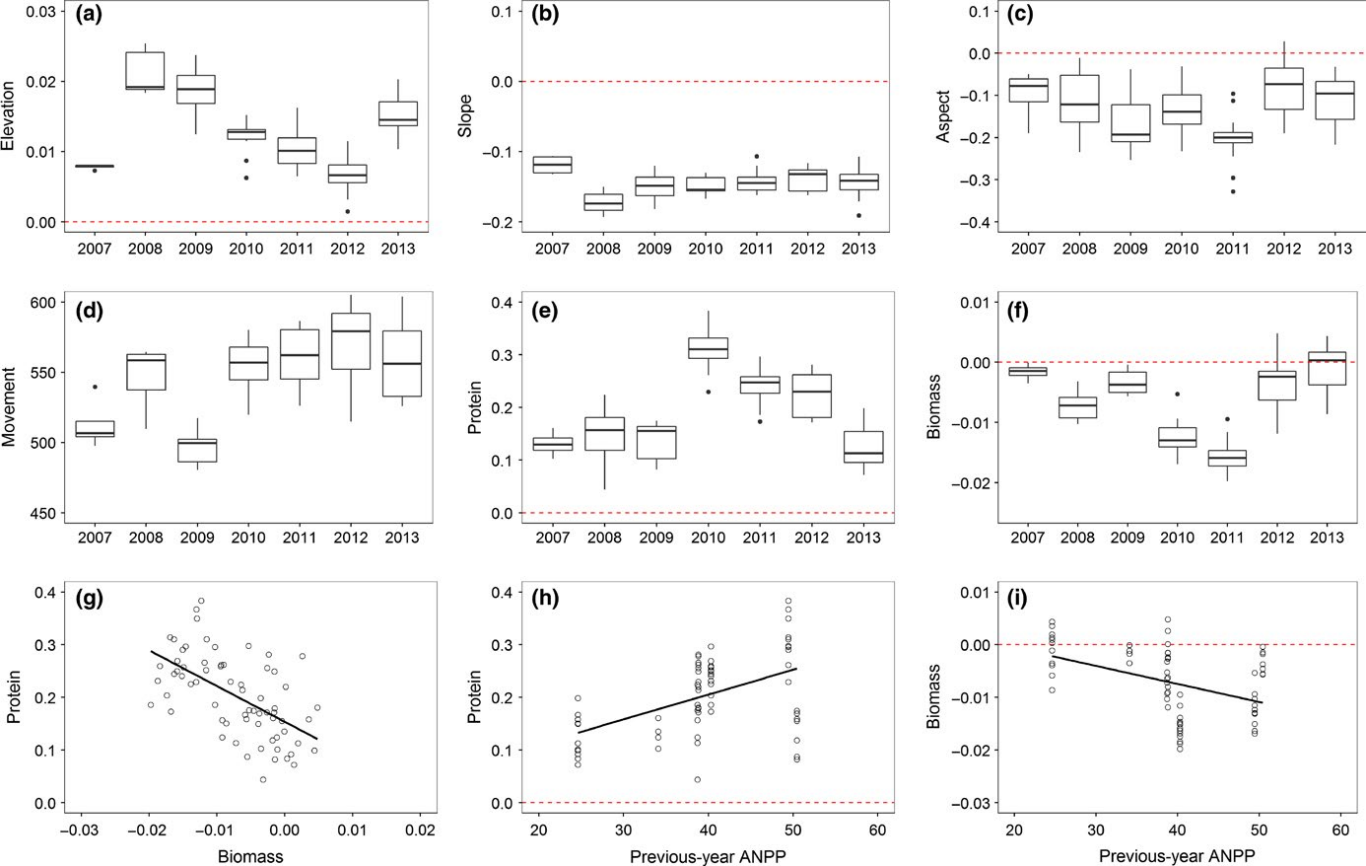
Boxplots of maximum‐likelihood parameter estimates ($\overset{¯}{\beta }$) of selection averaged among animals each year for (a) elevation, (b) slope, (c) cosine of aspect, (d) standard deviation of habitat‐independent movement kernel (m moved per 4‐hr fix), (e) grass crude protein content, (f) forage biomass, (g) linear relationship between individual animal selection (β) for foliar protein (%) and forage biomass (g m^2^), (h, i) represent relationship between the previous‐year ANPP (0.1 g m^2^) and the present‐year selection for foliar protein and forage biomass, respectively

**Figure A3:**
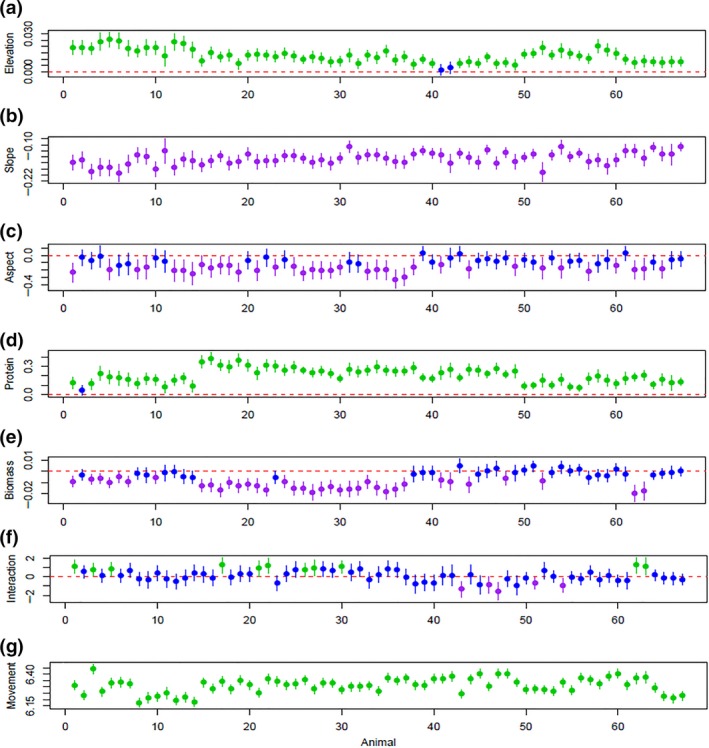
Maximum‐likelihood parameter estimates among all animals with mean (circle), 95% confidences (lines) and 0 (no selection) as the redline for (a) elevation, (b) slope, (c) cosine of aspect, (d) grass crude protein content, (e) forage biomass content, (f) protein–biomass interaction, and (g) habitat‐independent movement kernel parameter. Points that are green are positively associated with the variable, purple are negatively associated with the variable, and blue are not different from random

Bison exhibited habitat selection for all forage and topographic variables (Table A1), although variation in selection patterns among individuals was minimal based on random‐effects variance (range: 0 to 5E‐7; Table A2) and more substantial among years (Figure 3). Bison consistently exhibited selection for higher elevations (65 of 67 individual‐years) although preference differed among years (*F*_6, 51.28_ = 31.80, *p *< .0001; Figures 3a, A3a). In 2007 and 2012, strength of selection for elevation was lowest (Tukey's HSD test; *p* < .0001). Habitat selection coefficients associated with slope varied from 0 to −0.22 among all individuals and years (*F*_6,50.81_ = 5.81, *p* = .0001; Figures 3b and A3b), with strongest selection for slopes in 2007 and 2012, years following low forage production years (*p* ≤ .01). Preference for a southerly aspect was apparent in 36 of 67 individual‐years (54%; Figures 3c and A3c), while confidence intervals overlapped 0 for the other 31 individual‐years (*F*_6,53.6_ = 4.94, *p* = .0004). Variation in selection for southern aspect was evident across years with avoidance in 2011 being greater than 2012 and 2013 (*p *≤ .02).

The response to forage protein and biomass was more complex. All individual‐years except one favored habitat with high foliar protein content relative to available habitat (Figure A3d); however, the strength of selection for areas of foliar protein content varied among years (*F*_6,53.6_ = 27.71, *p* < .0001; Figure 3e). Selection strength for foliar protein was greatest from 2010 to 2011, years following high forage production years, and lowest in 2007 and 2013, years following low forage production years (*p *≤ .01). During the study, 36 of 67 individuals (54%) favored areas of lower herbaceous biomass during the growing season (Figure A3e). The strength of avoidance for areas of high herbaceous biomass content generally varied among years (*F*_6,52.27_ = 32.29, *p* < .0001; Figure 3f). Avoidance for areas of high forage biomass content was highest in 2008–2011, while selection for biomass in 2007 and 2012–2013 was not different from zero.

A significant interaction occurred between biomass and protein selection in 16 of 67 individual‐years (25%; Figure A3f). Variation in the forage quality–quantity interaction occurred across years with the 2008 (*n* = 3), 2010 (*n* = 3), 2011 (*n* = 7) interactions being significantly positive and significantly negative in 2012 (*n* = 3). There was consistent selection for higher protein (66 of 67), and some individuals avoided areas of higher biomass (36 of 67). The positive interaction between protein and biomass for 11 of these 36 animals implies high biomass is selected for when coupled with selection for high protein but not if selection for protein is low. Five other individuals showed no significant selection for biomass but showed negative interactions between protein and biomass, implying that these animals selected for areas of high protein and low biomass. There was no interaction between protein and biomass for 51 of 67 individual‐years, implying that the selection for biomass does not change with an individual's selection for protein. There was a single individual‐year in which there was no selection for protein, biomass, or their interaction. Overall, selection for herbaceous biomass was negatively correlated with selection for foliar crude protein content (LME; β ± SE = −6.78 ± 1.19, *p* < .0001; Figure 3g).

Visual inspection of three‐dimensional plots of probability of foliar protein and biomass habitat selection, where significant selection for these resources was inferred based on the confidence intervals not overlapping zero, showed individual‐level habitat selection strategies were composed of three forms, (1) selection for areas of high protein availability and areas of high and low levels of biomass (*n* = 34), (2) selection for areas of high protein availability but low biomass availability (*n* = 27), and (3) no significant selection for forage biomass (*n* = 6). Twenty‐five of the 41 (61%) lactating females with clear forage selection strategies exhibited the first strategy, while 11 of 20 (55%) nonlactating individuals selected for areas of high foliar protein availability but low forage content (Table A3).

A negative correlation between selection for higher elevation and growing season temperature was evident (LME; β ± SE = −0.003 ± 0.0003, *p* < .0001). Selection strength for slope was positively related to growing season temperature (0.01 ± 0.002, *p* = .004), while selection for southerly aspect was not related to growing season temperature (0.01 ± 0.01, *p* = .15). Selection for high foliar protein was positively related to growing season temperature (0.04 ± 0.01, *p* < .0001), while the relationship between selection for high forage biomass and growing season temperature was not significant (*p* = .17). A contrasting relationship of selection for protein and biomass with previous growing season ANPP was evident. Selection strength for protein was positively related to increasing previous growing season ANPP (β ± SE = 0.005 ± 0.001, *p* < .0001; Figure 3h), while selection strength for biomass was negatively related to increasing previous growing season ANPP (−0.0003 ± 0.0001, *p* = .0003; Figure 3i). Selection strength for topographic and forage attributes did not differ between lactating and nonlactating bison (*p* > .05). Local weather variables, previous‐year ANPP and current‐year growing season temperature, did not interact with reproductive status to explain selection strength for all grassland attribute variables (*p* > .05).

### 3.3 Movement

The standard deviations of the movement kernels, independent of the effect of habitat, were relatively consistent among animals, although some variation was observed among years (*F*_6,47.14_ = 13.43, *p* < .0001; Table A1). The standard deviation of the movement kernel ranged from 478 to 626 m (mean = 546 m), and the mean absolute displacement distances ranged from 383 to 498 m (mean = 436 m) in each 4 hr time step. In 2007 and 2009, the mean absolute displacement distances were lower than the other years in this study (513 and 496 m, respectively, compared to distances of 557–572 in the other years; *p* < .0001; Figure 3d).

## 4 Discussion

Understanding how movement patterns reflect animal interactions with their environment requires consideration of the temporally dynamic nature of those environments (Mueller et al., 2011; Owen‐Smith, Fryxell, & Merrill, 2010). In our study, bison movements were influenced by the spatial distribution and interyear variation in forage quality and quantity. Crude protein content of forage was a strong dynamic driver of resource selection across all summers. Allred, Fuhlendorf, Engle, and Elmore (2011) showed that crude protein content of graminoids is inversely related with time since fire in tallgrass prairie grazing systems, while forage quantity is positively related to time since fire. When infrequently burned tallgrass prairie is released from light limitation through prescribed burning in the presence of increased soil nutrients, prolonged availability of high‐quality forage is the result (Blair, 1997), and bison maintain the grassland in a state of low‐to‐intermediate biomass throughout the remainder of the growing season (Raynor et al., 2015). Foraging in these habitats allows large grazers to maintain their daily intake rate of digestible energy/protein (Bergman et al., 2001; Illius et al., 2002; Wilmshurst et al., 1995). Because stage of forage maturation is distributed variably across space and time, our approach of modeling biweekly changes in forage quality and quantity captured the spatiotemporal variation in response to prescribed burning and local weather. Thus, our findings describe the degree to which extrinsic factors modulate large grazer habitat selection in a fire‐prone grassland.

The relative strength of selection and relative avoidance of areas containing high crude protein content and high herbaceous biomass, respectively, varied from year to year. This indicates the magnitude of the forage quality–quantity tradeoff for large grazers varies in response to climatic conditions. Avoidance of areas with high herbaceous biomass coincided with high annual net primary productivity (ANPP), whereas avoidance of areas of high herbaceous biomass was weaker in years of low ANPP, thus meeting our first two predictions. Selection for foliar crude protein content was strongest during the moderately productive years of this study, weakest in above‐average forage production years, and moderate in low forage production years when available forage offers most protein content (Milchunas, Varnamkhasti, Lauenroth, & Goetz, 1995). Similar functional responses between resource selection and forage availability have been described in European cervids. Moderately abundant high‐quality forage has been shown to be the best predictor of habitat use in female roe deer (*Capreolus capreolus*) (Pellerin et al., 2010), while high‐quality forage is used less frequently when rare and habitat selection for high‐quality forage becomes saturated when it is abundant (Pellerin et al., 2010; Van Beest, Mysterud, Loe, & Milner, 2010).

Foliar protein concentration often increases in years of reduced precipitation (Joern & Mole, 2005; Jones & Coleman, 1991; Milchunas et al., 1995). Daily nutrient gains could be maximized by selecting areas containing small plants of high nutrient value as long as young forage tissue was available via regrowth (Augustine & Springer, 2013). Greater use of high foliar protein–low biomass habitat may allow large herbivores to maximize their summer dietary nitrogen intake and nutritional condition before entering winter (Hjeljord & Histol, 1999; McArt et al., 2009; Proffitt, Hebblewhite, Peters, Hupp, & Shamhart, 2016). For example, elk (*Cervus elaphus*) inhabiting summer ranges in western Montana, USA, with lower nutritional resources have lower nutritional condition entering winter which can result in lower pregnancy rates than elk inhabiting summer ranges with greater nutritional availability (Proffitt et al., 2016). Bison remember pertinent information about location and quality of forage resources across their landscape and may use this information to selectively move to areas of higher profitability (Merkle, Fortin, & Morales, 2014). Individual animals may favor the long‐term strategy of using areas where satiation may take longer to achieve, but more digestible nutrients may be attained, in largely predator‐free landscapes, such as Konza Prairie. Without the risk of predation, more time could be spent foraging instead of performing antipredator behavior (Creel, Schuette, & Christianson, 2014).

Our mechanistic movement modeling identified multiple abiotic features of the landscape that influenced movements of female bison. Selection coefficients for elevation were negatively related to increasing growing season temperatures (e.g., 2012), suggesting that bison may have been seeking water or shade in riparian areas at lower elevations in response to increased temperatures. Allred et al. (2013) showed large grazer attraction to low‐lying riparian areas in tallgrass prairie was strongest during days when operative temperature exceeded 29°C. Bison can also attain substantial amounts of water from wallows and forage after recent precipitation at KPBS (Nippert, Culbertson, Orozco, Ocheltree, & Helliker, 2013). We show movement decisions are related to elevation, and this relationship varied by growing season in response to local environmental conditions. The presence of steep slopes decreased the probability of selection, and bison generally avoided habitat that did not face south. Both of these behavioral responses to static environmental features could be fitness‐based. Locomotion on steep slopes increases energy expenditure as compared to level areas in ungulates (Dailey & Hobbs, 1989; Parker, Robbins, & Hanley, 1984). Some slopes in the bison enclosure are fairly steep, with areas of exposed soil and rock which reduce the probability that fire would cross and affect forage quality (Collins & Calabrese, 2012). Such slopes are less desirable to bison as foraging sites as the energetic demands required to utilize them may outweigh the benefits from the forage consumed. Selection for steeper slopes was strongest in years of low forage availability and high temperatures, which suggests steep slopes may prove useful as a forage reserve in periods of low food availability. Further, selection of nonsoutherly aspects was highest during the drought year (2012; Knapp et al., 2015), which corroborates the view that movement decisions during drought years may be primarily food driven; areas of nonsouthern aspect may provide more forage than the highly utilized, upland areas facing south. Topographic influences on soil moisture availability and grass productivity is a critical factor generating functional heterogeneity for herbivores during droughts because of the ability of more productive, wetter lowland parts of the landscape to produce reserves of forage during droughts (Augustine & Springer, 2013; Fynn, Augustine, Peel, & de Garine‐Wichatitsky, 2016; Hopcraft et al., 2010; Knapp et al., 1993). Although the magnitude of selection for topographic features does not necessarily translate into direct energy expenditure or gain, it does allow for comparison of the relative effects of different grassland attributes on movement decisions and providing insights into the fitness consequence of future environmental change (Mysterud, Yoccoz, Langvatn, Pettorelli, & Stenseth, 2008).

The majority of the bison in this study were lactating females that selected sites of high nutritional quality regardless of forage biomass levels (Table A3), likely so time spent locating forage could be minimized. Because energetic demands are greater in lactating females (Clutton‐Brock, Albon, & Guinness, 1989), bison could potentially meet their energetic requirements by selecting sites with high forage biomass when reducing satiation time is critical (i.e., to provide neonatal care) and also use low biomass sites providing accessibility to higher foliar protein (i.e., switching; Prins & Beekman, 1989). Adaptive foraging between a short, high‐quality grassland and a taller, lower quality grassland has been shown to result in greater overall intake and animal growth than for animals using only the short or tall grassland (Owen‐Smith, 2002; Prins & Beekman, 1989). This strategy was most common (68%) in bison that had raised calves in consecutive years (Table A3). In high forage production years, 2008–2009, 60% of consecutive‐year breeders employed this strategy while in 2012, a drought year, 86% engaged in this selection strategy; suggesting breeder selection strategies are climate‐dependent. A moderate correlation between previous‐year ANPP and forage resource selection indicated selection for forage availability was greater in years following low forage production than in years following high forage production. This trend was especially evident with lactating females. Apparently, selection decisions at the landscape scale for forage biomass, as observed only in years following low forage production years, allow large grazers to compensate for unfavorable temporal variation in resource availability (e.g., due to depletion of resources over the previous dormant season) (Fryxell et al., 2005; Hamel & Côté, 2008; Van Beest et al., 2010; Van der Wal et al., 2000). Our findings indicate that past growing season conditions may carry over to affect movement decisions in the following growing season.

Most nonlactating females in our study chose high forage quality sites where forage biomass was low. This behavior suggests a foraging strategy centered on site fidelity where returning to familiar patches can reduce time spent locating food (known high‐quality patches are easier to locate although they may offer less forage quantity; Merkle, Fortin, & Cherry, 2015; Schaefer, Bergman, & Luttich, 2000). This explanation is reasonable for nonlactating females with satiation requirements that are lower than lactating females (Clutton‐Brock et al., 1989).

Growing season movement rates were generally consistent across years with the exception of 2007 and 2009, when movement rates were lower than that observed in other years of this study. We surmise that the combined ideal rangeland conditions of average to below‐average temperature and above‐average rainfall during these growing seasons may be responsible (Pyke, Herrick, Shaver, & Pellant, 2002). Rather than spending more time seeking shade or water (Allred et al., 2013), large grazers can use this time to seek a more diverse diet (Bailey, Stephenson, & Pittarello, 2015).

The highly profitable uplands at KPBS provide suitable forage (e.g., *Bouteloua*), and the shallow, upland soils at KPBS offer plants of lower vegetative stature but of high protein content (Schimel et al. 1991). Increased nitrogen mineralization from additional nutrient inputs in the form of grazer excreta could positively affect vegetation growth rate and nutrient quality (Noy‐Meir, 1993). Strong selection for higher elevations in nondrought years suggests that movement is guided by high protein availability typical of the upper bench habitat when regrowth is possible. In years of low ANPP, bison distribution shifted from upper bench habitats to low elevation areas, where resources such as forage, water, and/or shade are available. Selection for areas of high biomass followed years of low ANPP, suggesting that lag effects of forage availability can impact animal movement. Our result that the strong selection for foliar protein in years following high ANPP implies that forage protein (nitrogen) content is a limiting resource that plays a critical yet overlooked role in driving large grazer distributions.

Extrinsic biases to bison movement were evaluated by comparing observed and random steps through the heterogeneous, fire‐prone landscape. Our procedure of simultaneously estimating the movement kernel and habitat preference models allows us to estimate intrinsic habitat preferences, independent of general movement (Avgar et al., 2016; Beyer et al., 2016; Forester et al., 2009; Prokopenko, Boyce, & Avgar, 2016). We apply this framework to a dynamic system with an intact fire–grazer interaction, which to our knowledge, is the first experimental evidence for demonstrating the variation in fine‐scale movement decisions dictated by forage resources under varying local climatic conditions in a fire‐prone system.

## 5 Conclusion

Resource‐driven movement patterns of bison in our experimental tallgrass prairie landscape are shaped by the forage quality–quantity tradeoff, site topography, and spatial distributions of resource availability. Although food quality is influential in resource selection and movement, understanding large grazer distribution and movement is multidimensional. This study provides a unique analysis of the role of forage dynamics and climate on the interannual variation of bison habitat selection. Our framework brings together the recent development of mechanistic movement models (Avgar et al., 2016; Beyer et al., 2016; Fortin et al., 2005; Prokopenko et al., 2016; Rhodes, McAlpine, Lunney, & Possingham, 2005) to quantify the effects of dynamic and static environmental variables on habitat selection for seven growing seasons.

In general, selection patterns reflected tradeoffs between individual goals (the need for accessible high‐quality forage in the postcalving period) and met our prediction for relative avoidance of areas of high forage biomass availability. Bison movements reflected the multiple biotic attributes of the landscape, which were variable from year to year and related to prevailing weather conditions. Step selection for areas of low‐to‐intermediate biomass explains patterns of uniform space use reported previously for large grazers in fire‐prone systems, where fire induces pulses in forage quality/accessibility and sets the stage for restricted space use of grazers in fire‐managed mesic grasslands for the rest of the growing season (Raynor et al. 2016; Vinton et al., 1993). Furthermore, individual‐level habitat selection varied little for all grassland attributes within years regardless of individual reproductive status, but the magnitude of selection varied substantially across years likely in response to weather conditions and concomitant forage quality and quantity. Our finding that individual habitat selection behavior does not depend on reproductive status combined with the similarity in habitat selection behavior expressed by individual bison indicates that group‐fusion dynamics are strong drivers of habitat selection and not intrinsic factors such as reproductive status.

Understanding how forage quality–quantity tradeoffs drive large grazer habitat use in the Great Plains is critical to sustainable rangeland management. Warming and drying are anticipated to reduce plant production and nutritive content in the southern Great Plains (Briske et al., 2015). These changes are likely to negatively affect rangeland economics by reducing stocking rates and total livestock production (Polley et al., 2013). Our findings provide insight into how a large grazer selects habitat in growing seasons of varying resource availability due to local climate conditions. For example, we found step selection for high elevation to be lowest in drought conditions. This finding indicates that during droughts burning lowlands due to their higher soil moisture availability and not burning uplands that are incapable of providing high‐quality regrowth may provide a means for restricting space use as well as reducing land degradation and thus optimize animal protein intake and land use (Fynn et al., 2016; Knapp et al., 1993; Parrini & Owen‐Smith, 2009).

While grassland fires can have pronounced effects on landscape‐scale distributions of large herbivores (Allred et al., 2011; Sensenig, Demment, & Laca, 2010), the underlying dynamic forage resources directly responsible for these distributions are largely unexplored. Our data suggest that fire‐induced heterogeneity coupled with climatic responses in vegetation quality are an important landscape‐scale process that helps promote nutrient attainment in large herbivores and illustrates the utility of linking foraging theory with insights from resource and movement ecology. Our analyses are a critical but rarely documented aspect of understanding this connection between resource use and population ecology.

## Conflict of Interest

None declared.

## Appendix 1

### Herd History and Management

The KPBS bison herd was established in 1987 and is currently maintained at a stocking level of ~260 adult individuals, with ~80 calves born each spring. Bison (identifiable by uniquely numbered ear tags) are weighed, and their general health assessed at an annual roundup of all animals in late October/early November; some individuals are culled at this time to maintain prescribed stocking densities. Young animals (~2 years of age or yearlings), old animals, and excess males are removed from the herd resulting in a sex ratio of mature females to mature males of approximately 4:1. All males >8 years are removed, while females may remain until the age of 15+ years (Ungerer, Weitekamp, Joern, Towne, & Briggs, 2013).

**Table A1:** Maximum‐likelihood parameter estimates (*x*′ ± 95% CI) among all animals and years for elevation, slope, cosine of aspect, grass crude protein content, herbaceous biomass content, the interaction of foliar protein and biomass, and movement from 2007 to 2013 at KPBS

ID	Year	Elevation	Slope	Cos(Aspect)	Protein	Biomass	Protein × Biomass	Movement
w514	2007	0.01 (0, 0.01)	−0.13 (−0.17, −0.1)	−0.06 (−0.19, 0.06)	0.12 (0.05, 0.2)	0 (−0.01, 0)	−0.18 (−0.83, 0.47)	6.21 (6.17, 6.25)
w514	2008	0.02 (0.01, 0.02)	−0.16 (−0.18, −0.13)	−0.23 (−0.37, −0.1)	0.12 (0.06, 0.19)	−0.01 (−0.01, 0)	1.09 (0.39, 1.79)	6.31 (6.27, 6.35)
w531	2008	0.02 (0.01, 0.02)	−0.15 (−0.18, −0.12)	−0.03 (−0.14, 0.08)	0.04 (−0.01, 0.09)	0 (−0.01, 0)	0.53 (−0.11, 1.17)	6.23 (6.2, 6.27)
w531	2009	0.02 (0.01, 0.02)	−0.13 (−0.16, −0.11)	−0.19 (−0.32, −0.06)	0.12 (0.06, 0.18)	0 (−0.01, 0)	−0.25 (−0.95, 0.45)	6.17 (6.14, 6.21)
w531	2010	0.01 (0.01, 0.02)	−0.16 (−0.18, −0.13)	−0.2 (−0.34, −0.07)	0.23 (0.16, 0.3)	−0.01 (−0.02, −0.01)	0.89 (0.01, 1.77)	6.25 (6.22, 6.29)
w630	2009	0.02 (0.01, 0.03)	−0.14 (−0.16, −0.11)	−0.16 (−0.32, −0.01)	0.17 (0.11, 0.23)	0 (−0.01, 0)	−0.31 (−1.21, 0.59)	6.21 (6.17, 6.26)
w651	2008	0.02 (0.01, 0.02)	−0.19 (−0.22, −0.16)	−0.07 (−0.19, 0.04)	0.11 (0.06, 0.17)	−0.01 (−0.01, 0)	0.77 (0.12, 1.43)	6.44 (6.4, 6.48)
w651	2009	0.02 (0.01, 0.02)	−0.18 (−0.21, −0.16)	−0.04 (−0.17, 0.09)	0.16 (0.1, 0.22)	−0.01 (−0.01, 0)	0.36 (−0.44, 1.15)	6.22 (6.19, 6.26)
w651	2010	0.01 (0.01, 0.02)	−0.15 (−0.17, −0.13)	−0.03 (−0.15, 0.09)	0.31 (0.25, 0.37)	−0.02 (−0.02, −0.01)	1.2 (0.37, 2.03)	6.36 (6.33, 6.4)
w651	2011	0.01 (0, 0.01)	−0.14 (−0.17, −0.12)	−0.19 (−0.33, −0.04)	0.2 (0.15, 0.26)	−0.02 (−0.03, −0.01)	1.12 (0.19, 2.05)	6.37 (6.33, 6.42)
w753	2007	0.01 (0, 0.01)	−0.13 (−0.15, −0.11)	−0.19 (−0.31, −0.07)	0.16 (0.1, 0.22)	0 (−0.01, 0)	−0.17 (−0.82, 0.48)	6.23 (6.19, 6.26)
w753	2008	0.02 (0.02, 0.03)	−0.17 (−0.2, −0.15)	−0.01 (−0.16, 0.13)	0.22 (0.15, 0.3)	−0.01 (−0.01, 0)	0.13 (−0.6, 0.87)	6.26 (6.22, 6.3)
w753	2009	0.01 (0, 0.02)	−0.12 (−0.16, −0.08)	−0.08 (−0.23, 0.07)	0.08 (0.02, 0.14)	0 (−0.01, 0.01)	−0.21 (−1.11, 0.68)	6.25 (6.2, 6.29)
w764	2008	0.03 (0.02, 0.03)	−0.18 (−0.2, −0.15)	−0.19 (−0.33, −0.05)	0.19 (0.12, 0.25)	−0.01 (−0.01, −0.01)	0.85 (0.14, 1.55)	6.33 (6.29, 6.37)
w764	2009	0.02 (0.02, 0.03)	−0.17 (−0.2, −0.15)	−0.21 (−0.35, −0.06)	0.16 (0.1, 0.21)	0 (−0.01, 0.01)	−0.52 (−1.34, 0.3)	6.19 (6.16, 6.23)
w764	2010	0.01 (0.01, 0.02)	−0.15 (−0.17, −0.13)	−0.16 (−0.28, −0.04)	0.3 (0.24, 0.36)	−0.01 (−0.01, 0)	−0.66 (−1.46, 0.14)	6.34 (6.3, 6.38)
w764	2011	0.01 (0.01, 0.01)	−0.15 (−0.16, −0.13)	−0.25 (−0.34, −0.15)	0.26 (0.22, 0.3)	−0.01 (−0.02, −0.01)	0.77 (0.04, 1.5)	6.35 (6.32, 6.39)
w764	2012	0.01 (0, 0.01)	−0.13 (−0.15, −0.11)	−0.16 (−0.27, −0.05)	0.28 (0.22, 0.34)	0 (−0.01, 0)	−0.76 (−1.77, 0.25)	6.31 (6.28, 6.35)
w764	2013	0.01 (0.01, 0.02)	−0.14 (−0.16, −0.13)	−0.06 (−0.16, 0.04)	0.09 (0.05, 0.13)	0 (0, 0.01)	−0.15 (−0.78, 0.48)	6.28 (6.24, 6.31)
y026	2010	0.01 (0, 0.01)	−0.17 (−0.19, −0.14)	−0.13 (−0.26, 0)	0.35 (0.28, 0.41)	−0.01 (−0.02, −0.01)	0.27 (−0.59, 1.12)	6.33 (6.3, 6.37)
y026	2011	0.01 (0.01, 0.02)	−0.16 (−0.18, −0.14)	−0.2 (−0.32, −0.08)	0.23 (0.18, 0.28)	−0.02 (−0.03, −0.01)	0.95 (0.09, 1.81)	6.28 (6.25, 6.32)
y026	2012	0.01 (0.01, 0.02)	−0.12 (−0.13, −0.1)	−0.04 (−0.14, 0.05)	0.22 (0.18, 0.26)	0 (−0.01, 0.01)	−0.89 (−1.76, −0.01)	6.3 (6.27, 6.34)
y026	2013	0.01 (0.01, 0.02)	−0.13 (−0.15, −0.12)	−0.1 (−0.19, 0)	0.1 (0.06, 0.14)	0 (0, 0.01)	−0.72 (−1.36, −0.08)	6.28 (6.25, 6.32)
y036	2010	0.02 (0.01, 0.02)	−0.15 (−0.18, −0.13)	−0.17 (−0.3, −0.05)	0.38 (0.32, 0.45)	−0.01 (−0.02, −0.01)	−0.12 (−1, 0.76)	6.29 (6.25, 6.32)
y036	2011	0.01 (0.01, 0.01)	−0.15 (−0.17, −0.13)	−0.2 (−0.32, −0.09)	0.25 (0.2, 0.3)	−0.02 (−0.02, −0.01)	0.81 (0, 1.62)	6.33 (6.3, 6.37)
y036	2012	0.01 (0.01, 0.01)	−0.12 (−0.13, −0.1)	0.03 (−0.07, 0.13)	0.18 (0.14, 0.22)	0 (−0.01, 0)	−0.61 (−1.49, 0.27)	6.31 (6.28, 6.35)
y072	2010	0.01 (0.01, 0.02)	−0.14 (−0.16, −0.12)	−0.14 (−0.25, −0.02)	0.31 (0.25, 0.37)	−0.02 (−0.02, −0.01)	1.25 (0.41, 2.09)	6.34 (6.31, 6.38)
y072	2011	0.01 (0, 0.01)	−0.12 (−0.14, −0.1)	−0.2 (−0.32, −0.07)	0.19 (0.14, 0.23)	−0.02 (−0.03, −0.01)	1.25 (0.4, 2.1)	6.37 (6.33, 6.41)
y072	2012	0.01 (0, 0.01)	−0.16 (−0.18, −0.14)	−0.08 (−0.18, 0.02)	0.28 (0.22, 0.34)	0 (0, 0.01)	−1.55 (−2.53, −0.58)	6.4 (6.37, 6.44)
y116	2010	0.01 (0.01, 0.02)	−0.16 (−0.18, −0.14)	−0.14 (−0.26, −0.02)	0.3 (0.23, 0.36)	−0.01 (−0.02, 0)	−0.04 (−0.83, 0.75)	6.28 (6.25, 6.32)
y116	2011	0.01 (0, 0.01)	−0.16 (−0.18, −0.14)	−0.21 (−0.32, −0.1)	0.23 (0.18, 0.27)	−0.01 (−0.02, −0.01)	0.69 (−0.08, 1.45)	6.33 (6.3, 6.37)
y116	2012	0.01 (0, 0.01)	−0.13 (−0.15, −0.11)	−0.09 (−0.19, 0.01)	0.17 (0.13, 0.21)	0 (−0.01, 0)	−0.66 (−1.52, 0.21)	6.36 (6.33, 6.4)
y116	2013	0.02 (0.01, 0.02)	−0.19 (−0.22, −0.16)	−0.18 (−0.33, −0.03)	0.15 (0.08, 0.22)	−0.01 (−0.02, 0)	0.64 (−0.27, 1.55)	6.28 (6.24, 6.31)
y139	2007	0.01 (0, 0.01)	−0.11 (−0.12, −0.09)	−0.05 (−0.15, 0.05)	0.14 (0.1, 0.17)	0 (0, 0)	−0.33 (−0.91, 0.26)	6.23 (6.19, 6.26)
y139	2008	0.02 (0.02, 0.03)	−0.19 (−0.22, −0.17)	−0.13 (−0.27, 0.01)	0.18 (0.1, 0.25)	−0.01 (−0.01, 0)	0.1 (−0.62, 0.81)	6.34 (6.3, 6.37)
y139	2010	0.01 (0, 0.01)	−0.16 (−0.18, −0.13)	−0.23 (−0.36, −0.11)	0.37 (0.3, 0.43)	−0.01 (−0.02, −0.01)	0.34 (−0.5, 1.18)	6.35 (6.31, 6.39)
y139	2011	0.01 (0, 0.01)	−0.14 (−0.16, −0.12)	−0.16 (−0.27, −0.06)	0.17 (0.13, 0.21)	−0.02 (−0.02, −0.01)	1.07 (0.38, 1.76)	6.28 (6.24, 6.31)
y139	2012	0.01 (0, 0.01)	−0.13 (−0.14, −0.11)	−0.04 (−0.13, 0.06)	0.21 (0.17, 0.25)	−0.01 (−0.01, 0)	−0.2 (−1.03, 0.64)	6.41 (6.37, 6.44)
y139	2013	0.01 (0.01, 0.02)	−0.13 (−0.15, −0.12)	−0.03 (−0.13, 0.07)	0.1 (0.06, 0.14)	0 (−0.01, 0)	0 (−0.65, 0.64)	6.27 (6.23, 6.3)
y269	2009	0.02 (0.02, 0.03)	−0.15 (−0.17, −0.13)	−0.21 (−0.36, −0.06)	0.17 (0.12, 0.23)	0 (−0.01, 0)	−0.13 (−1, 0.73)	6.22 (6.18, 6.25)
y269	2010	0.01 (0.01, 0.02)	−0.14 (−0.16, −0.12)	−0.06 (−0.18, 0.06)	0.26 (0.2, 0.32)	−0.01 (−0.01, 0)	0.34 (−0.47, 1.15)	6.31 (6.28, 6.35)
y269	2011	0.01 (0.01, 0.02)	−0.11 (−0.13, −0.09)	−0.1 (−0.23, 0.04)	0.27 (0.2, 0.33)	−0.02 (−0.02, −0.01)	0.5 (−0.4, 1.4)	6.3 (6.27, 6.34)
y269	2013	0.02 (0.01, 0.02)	−0.11 (−0.13, −0.08)	−0.18 (−0.31, −0.05)	0.16 (0.09, 0.22)	0 (0, 0.01)	−0.95 (−1.72, −0.18)	6.33 (6.3, 6.37)
y270	2010	0.01 (0.01, 0.02)	−0.13 (−0.15, −0.11)	−0.07 (−0.19, 0.05)	0.31 (0.25, 0.37)	−0.01 (−0.02, −0.01)	0.27 (−0.51, 1.05)	6.31 (6.28, 6.35)
y270	2011	0.01 (0, 0.01)	−0.14 (−0.16, −0.12)	−0.11 (−0.23, 0.01)	0.24 (0.18, 0.3)	−0.02 (−0.02, −0.01)	0.81 (−0.01, 1.63)	6.3 (6.26, 6.34)
y270	2012	0.01 (0, 0.01)	−0.16 (−0.18, −0.13)	−0.15 (−0.27, −0.03)	0.25 (0.18, 0.32)	0 (−0.01, 0.01)	−0.92 (−1.99, 0.14)	6.34 (6.3, 6.37)
y270	2013	0.01 (0.01, 0.02)	−0.14 (−0.16, −0.12)	−0.08 (−0.18, 0.02)	0.08 (0.05, 0.12)	0 (0, 0.01)	−0.04 (−0.67, 0.59)	6.27 (6.24, 6.31)
y274	2007	0.01 (0, 0.01)	−0.11 (−0.12, −0.09)	−0.09 (−0.19, 0.01)	0.1 (0.07, 0.14)	0 (−0.01, 0)	0.18 (−0.38, 0.74)	6.29 (6.25, 6.33)
y274	2008	0.02 (0.01, 0.02)	−0.16 (−0.19, −0.14)	−0.12 (−0.27, 0.03)	0.16 (0.08, 0.23)	−0.01 (−0.01, 0)	0.7 (−0.04, 1.43)	6.33 (6.29, 6.36)
y274	2009	0.02 (0.01, 0.02)	−0.15 (−0.18, −0.12)	−0.25 (−0.41, −0.1)	0.09 (0.03, 0.15)	−0.01 (−0.01, 0)	0.42 (−0.39, 1.24)	6.18 (6.14, 6.22)
y274	2010	0.01 (0.01, 0.02)	−0.14 (−0.16, −0.11)	−0.15 (−0.28, −0.03)	0.29 (0.23, 0.35)	−0.02 (−0.02, −0.01)	0.78 (−0.09, 1.66)	6.32 (6.28, 6.36)
y274	2011	0.01 (0.01, 0.02)	−0.13 (−0.15, −0.11)	−0.21 (−0.33, −0.09)	0.26 (0.2, 0.31)	−0.01 (−0.02, 0)	−0.35 (−1.2, 0.5)	6.31 (6.28, 6.35)
y274	2012	0 (0, 0.01)	−0.13 (−0.16, −0.11)	−0.13 (−0.25, −0.02)	0.23 (0.16, 0.3)	−0.01 (−0.01, 0)	0.11 (−0.86, 1.07)	6.36 (6.33, 6.4)
y274	2013	0.01 (0.01, 0.02)	−0.13 (−0.14, −0.11)	−0.07 (−0.17, 0.03)	0.07 (0.04, 0.11)	0 (0, 0.01)	−0.26 (−0.89, 0.37)	6.37 (6.33, 6.41)
y389	2012	0 (0, 0.01)	−0.16 (−0.19, −0.13)	−0.04 (−0.17, 0.1)	0.26 (0.19, 0.34)	−0.01 (−0.02, 0)	0.14 (−0.98, 1.26)	6.38 (6.35, 6.42)
y389	2013	0.01 (0.01, 0.01)	−0.16 (−0.18, −0.13)	−0.22 (−0.34, −0.09)	0.17 (0.11, 0.23)	−0.01 (−0.01, 0)	0.45 (−0.31, 1.22)	6.35 (6.32, 6.39)
y507	2013	0.02 (0.02, 0.03)	−0.15 (−0.17, −0.13)	−0.11 (−0.24, 0.01)	0.2 (0.14, 0.26)	0 (−0.01, 0)	−0.29 (−1.04, 0.45)	6.32 (6.29, 6.36)
y520	2011	0.01 (0.01, 0.02)	−0.13 (−0.15, −0.11)	−0.19 (−0.32, −0.06)	0.3 (0.23, 0.36)	−0.01 (−0.02, −0.01)	0.24 (−0.64, 1.12)	6.27 (6.23, 6.3)
y520	2012	0.01 (0, 0.01)	−0.13 (−0.14, −0.11)	0.02 (−0.08, 0.12)	0.18 (0.14, 0.22)	0 (0, 0.01)	−1.33 (−2.19, −0.47)	6.24 (6.21, 6.28)
y605	2011	0.02 (0.01, 0.02)	−0.15 (−0.17, −0.12)	−0.2 (−0.33, −0.07)	0.26 (0.21, 0.31)	−0.02 (−0.03, −0.01)	0.88 (0, 1.76)	6.37 (6.34, 6.41)
y605	2012	0.01 (0, 0.01)	−0.14 (−0.16, −0.12)	−0.19 (−0.31, −0.07)	0.27 (0.2, 0.33)	−0.01 (−0.02, 0)	0.24 (−0.8, 1.28)	6.36 (6.33, 6.4)
y605	2013	0.02 (0.01, 0.02)	−0.17 (−0.2, −0.14)	−0.06 (−0.19, 0.08)	0.15 (0.09, 0.21)	0 (−0.01, 0)	0.1 (−0.62, 0.83)	6.38 (6.35, 6.42)
y678	2011	0.01 (0.01, 0.01)	−0.16 (−0.18, −0.13)	−0.33 (−0.45, −0.2)	0.24 (0.2, 0.29)	−0.02 (−0.02, −0.01)	0.72 (−0.07, 1.51)	6.35 (6.31, 6.38)
y678	2012	0.01 (0.01, 0.01)	−0.12 (−0.14, −0.1)	0.03 (−0.07, 0.13)	0.17 (0.11, 0.24)	0 (−0.01, 0)	−0.43 (−1.35, 0.49)	6.31 (6.28, 6.35)
y720	2011	0.01 (0.01, 0.02)	−0.16 (−0.18, −0.14)	−0.3 (−0.42, −0.17)	0.25 (0.2, 0.3)	−0.01 (−0.02, −0.01)	−0.03 (−0.85, 0.8)	6.37 (6.33, 6.4)
y720	2012	0.01 (0, 0.01)	−0.16 (−0.18, −0.14)	−0.07 (−0.18, 0.03)	0.26 (0.2, 0.31)	0 (−0.01, 0)	−0.84 (−1.82, 0.13)	6.41 (6.37, 6.44)
y720	2013	0.01 (0.01, 0.02)	−0.15 (−0.17, −0.13)	−0.14 (−0.24, −0.04)	0.11 (0.06, 0.16)	0 (0, 0.01)	−0.43 (−1.09, 0.24)	6.4 (6.37, 6.44)

**Table A2:** Individual ID and residual variance (SD) of linear mixed models for grassland attributes and independent variables for bison resource selection at Konza Prairie Biological Station, Kansas, USA

Selection variable	Independent variable	Individual ID variance	Residual variance
Protein	Year (categorical)	2.03E‐18 (4.51E‐10)	1.71E‐3 (4.13E‐2)
Biomass	Year (categorical)	8.61E‐7 (9.23E‐4)	1.09E‐5 (3.30E‐3)
Elevation	Year (categorical)	1.11E‐6 (1.06E‐3)	2.63E‐3 (2.63E‐3)
Slope	Year (categorical)	5.10E‐5 (7.14E‐3)	2.48E‐4 (1.57E‐2)
Cosine Aspect	Year (categorical)	1.00E‐7 (1.0E‐6)	4.57E‐3 (6.76E‐2)
Protein	Previous‐year ANPP (0.1 g m^2^)	3.46E‐4 (1.86E‐2)	4.23E‐3 (6.51E‐2)
Biomass	Previous‐year ANPP (0.1 g m^2^)	1.0E‐9 (1.0E‐7)	3.61E‐5 (6.01E‐3)
Elevation	Previous‐year ANPP (0.1 g m^2^)	1.0E‐9 (1.0E‐7)	3.0E‐5 (5.46E‐3)
Slope	Previous‐year ANPP (0.1 g m^2^)	1.73E‐5 (4.16E‐3)	3.92E‐4 (1.98E‐2)
Cosine Aspect	Previous‐year ANPP (0.1 g m^2^)	1.0E‐7 (1.0E‐6)	6.09E‐3 (7.80E‐2)
Protein	Growing Season Temperature (°C)	2.83E‐20 (5.32E‐10)	3.97E‐3 (6.30E‐2)
Biomass	Growing Season Temperature (°C)	1.59E‐20 (1.26E‐10)	4.30E‐5 (6.56E‐3)
Elevation	Growing Season Temperature (°C)	1.0E‐10 (1.01E‐7)	1.10E‐5 (3.31E‐3)
Slope	Growing Season Temperature (°C)	3.20E‐5 (5.66E‐3)	3.34E‐4 (1.83E‐2)
Cosine Aspect	Growing Season Temperature (°C)	1.29E‐20 (3.59E‐10)	6.12E‐3 (7.83E‐2)

**Table A3:** Dynamic resource selection strategy of 20 female bison, their age, and if they reared a calf that year (0 or 1) or in two consecutive years at Konza Prairie Biological Station, Manhattan, Kansas, USA from 2007 to 2013. Strategies were determined from examining three‐dimensional plots of the probability of selection (*z*‐axis) over biomass (*x*), and foliar protein (*y*). (NA) data not available, (+) probability of selection is positive, (−) probability of selection is negative, (0) probability of selection is nondirectional

ID	Year	Age	Calf	Consecutive‐year calf	High protein	Low protein	High biomass	Low biomass
w514	2007	12	1	NA	+	−	−	+
w514	2008	13	0	0	+	−	+	+
w531	2008	13	1	0	+	−	+	+
w531	2009	14	0	0	+	−	+	+
w531	2010	15	1	0	+	−	−	+
w630	2009	13	0	0	+	−	+	+
w651	2008	12	1	1	+	−	−	+
w651	2009	13	1	1	+	−	−	+
w651	2010	14	1	1	+	−	−	+
w651	2011	15	0	0	+	−	−	+
w753	2007	10	0	NA	+	−	+	+
w753	2008	11	1	0	+	−	−	+
w753	2009	12	0	0	+	−	+	+
w764	2008	11	1	1	+	−	+	+
w764	2009	12	1	1	+	−	−	+
w764	2010	13	1	1	+	−	+	+
w764	2011	14	0	0	+	0	+	0
w764	2012	15	1	0	+	−	+	+
w764	2013	16	1	1	+	−	+	+
y026	2010	10	1	1	+	−	+	+
y026	2011	11	1	1	+	−	−	+
y026	2012	12	0	0	+	−	+	+
y026	2013	13	1	0	+	−	−	+
y036	2010	10	1	1	+	−	+	+
y036	2011	11	0	0	+	−	−	+
y036	2012	12	1	0	+	0	−	0
y072	2010	10	0	0	+	−	−	+
y072	2011	11	1	0	+	−	+	+
y072	2012	12	0	0	+	−	−	+
y116	2010	9	1	1	+	−	+	+
y116	2011	10	0	0	+	−	−	+
y116	2012	11	1	0	+	−	+	+
y116	2013	12	0	0	+	−	+	+
y139	2007	6	1	NA	+	−	−	+
y139	2008	7	1	1	+	−	+	+
y139	2010	9	1	1	+	−	+	+
y139	2011	10	1	1	+	−	0	−
y139	2012	11	0	0	+	−	−	+
y139	2013	12	1	0	+	−	−	+
y269	2009	7	1	1	+	−	−	+
y269	2010	8	0	0	+	−	+	+
y269	2011	9	1	0	+	−	−	+
y269	2013	11	1	0	+	−	+	+
y270	2010	8	1	1	+	−	+	+
y270	2011	9	0	0	+	−	+	+
y270	2012	10	1	0	+	−	+	+
y270	2013	11	0	0	+	−	−	+
y274	2007	5	1	NA	+	−	+	+
y274	2008	6	1	1	+	−	+	+
y274	2009	7	1	1	+	−	+	+
y274	2010	8	1	1	+	−	−	+
y274	2011	9	1	1	+	−	+	+
y274	2012	10	1	1	+	−	+	+
y274	2013	11	0	0	+	−	−	+
y389	2012	9	1	0	+	−	+	+
y389	2013	10	1	1	+	−	0	−
y507	2013	8	0	0	+	−	−	+
y520	2011	6	1	0	+	0	−	0
y520	2012	7	0	0	+	−	−	+
y605	2011	5	1	0	+	−	−	+
y605	2012	6	1	1	+	−	+	+
y605	2013	7	1	1	+	−	+	+
y678	2011	5	1	0	+	−	+	+
y678	2012	6	0	0	+	−	−	+
y720	2011	4	1	0	+	−	−	+
y720	2012	5	0	0	+	0	−	0
y720	2013	6	1	0	+	−	=	+
